# Study of the Thermal Decomposition Process of Explosive Mixtures Based on Hydrogen Peroxide

**DOI:** 10.3390/molecules29235616

**Published:** 2024-11-27

**Authors:** Roman Zakusylo, Oksana Pavlenko, Tomasz Jarosz, Andrzej Maranda, Daryna Zakusylo, Agnieszka Stolarczyk

**Affiliations:** 1 Shostka Institute, Sumy State University, 41100 Shostka, Ukraine; r.zakusylo@ishostka.sumdu.edu.ua (R.Z.); o.pavlenko@ishostka.sumdu.edu.ua (O.P.); d.zakusylo@ishostka.sumdu.edu.ua (D.Z.); 2 Department of Physical Chemistry and Technology of Polymers, Silesian University of Technology, 44-100 Gliwice, Poland; 3 Łukasiewicz Research Network, Institute of Industrial Organic Chemistry, 03-236 Warsaw, Poland; andrzej.maranda@ipo.lukasiewicz.gov.pl

**Keywords:** hydrogen peroxide, explosive, glass microspheres, thermal analysis

## Abstract

In this work, we have investigated the thermal features of hydrogen peroxide-based energetic materials formulations. Initial research has shown that both the auxiliary oxidiser (sodium nitrate, potassium nitrate or calcium nitrate) and sensitising agent (glass microspheres) have significant influence on the rate of hydrogen peroxide decay in such formulations. In terms of the thermal features of the tested energetic materials, a similar and significant influence of the auxiliary oxidising agent and sensitising agent choice was observed. We have established that the use of calcium nitrate as an auxiliary oxidising agent (at ambient temperature of approx 20 °C), which allows the formulations to maintain capacity to undergo detonation for longer under storage conditions, negatively impacts the qualitative characteristics of the mixture as an energetic material. The thermal effects accompanying chemical interaction are much smaller than mixtures containing potassium and sodium nitrates as additional oxidising agents. Another important conclusion is that glass microspheres as sensitising agents significantly impact the thermal decomposition processes of the investigated on-site mixed (OSM) energetic material (EM) samples, except for the mixture using calcium nitrate.

## 1. Introduction

Concentrated (i.e., aqueous solutions of hydrogen peroxide, whose concentration exceeds 50 wt. %), also known as high-test, hydrogen peroxide (HTP), has been known as a component of energetic material (EM) formulations [1] since the invention of liquid HTP-based EMs [2] and such EMs have been studied to an extent [3]. That research resulted in a number of publications, as well as patents on the subject [4]. These works primarily focused on the detonability of binary and ternary mixtures of HTP with various liquid fuels. This narrow focus brought about a temporary decline in the research interest dedicated to HTP-based EMs, primarily due to the constraints involved with the handling and use of liquid EMs.

Recently, however, interest in HTP-based EMs has seen a significant uptick [5], which was largely caused by the development of gel-like EM formulations. This increase in attention is further boosted by the drawbacks of many traditional EMs, particularly their harsh adverse impact on both human health and the environment [6,7,8,9,10]. In contrast, HTP is perceived as a “green” oxidising agent and a potential replacement for hazardous and harmful nitrocompounds and nitric acid esters [11,12,13,14,15,16]. The use of HTP in EMs leads to formulations that emit no nitrogen oxides upon detonation [17]. Another significant advantage of the most recent generation of HTP-based EMs is that they can be produced on site, via mixing (OSM-type EMs), from non-explosive and non-toxic components, providing drastically increased workplace safety. Moreover, due to the gradual decay of HTP within OSM-type EMs, any produced charges eventually lose their ability to detonate, mitigating the risks associated with misfires and with theft of EMs by unlawful parties.

Despite the increased interest in and advantages of OSM-type EMs, this class of EM formulations remains drastically under-researched. Apart from our work, no information is available on the decay of HTP (and, therefore, on the loss of energetic parameters) within HTP-based EMs over time [18]. Moreover, no information has been reported on the thermal features of such EM formulations. While an assumption can be made that increased temperature will promote HTP decay, it also influences the viscosity of the entire formulation as well as the interactions between its components, leading to a non-straightforward relationship. Consequently, in this study, we have aimed to present an accurate and comprehensive description of the thermal decomposition processes taking place in HTP-bearing OSM-type EMs.

Instead of using ammonium nitrate, which was found to drastically increase the HTP decay rate, we have opted to use less active auxiliary oxidising agents, i.e., calcium nitrate (CN), potassium nitrate (PN), and sodium nitrate (SN). Based on previous research, guar gum was selected as the thickener, as it is likely to have the optimal composition in terms of energetic properties. In this work, we have also focused heavily on studying the impact of the sensitising agent, i.e., glass microspheres (MS) on the thermal decomposition of OSM-type EMs. This is tied to our previously reported discovery of the significant impact of the type of microsphere on the rate of decay of HTP [18].

The use of sensitising additives in EMs started in the 1950s, with the detailed description of theories of localised initiation centres as part of the detonation process [19]. The postulated occurrence of localised heating in EMs under the influence of applied energy explains the increased susceptibility of chemical substances to detonation. These localised heating zones have various definitions: “hot spots, energy epicenters, or hot points”. The increasing prevalence of these thermal centres in the EM results in the intensification of the reaction of the EM to a potential detonation and the increased potential of thermal centres is reflected as an increased sensitivity to detonation for such EMs.

Frictional heating can be caused by the contact of moving particles from impact, inducing a flow of liquid or liquid-like EMs. Examples of additives considered beneficial for this mechanism include carborundum sand and similar materials with a melting temperature higher than 400 °C and low thermal conductivity. One version of this theory suggests that frictional heating is concentrated at the irregular points of the foreign particle surfaces in a sufficiently large manner to cause localised decomposition from the heat-initiated reaction, in turn causing local shock waves, further encouraging the addition of friction and so on until the detonation of the entire liquid. Similarly, adiabatic heating, caused by the compression of tiny gas bubbles, triggers thermal decomposition reactions of the energetic material at the relevant critical temperature, which produce shock waves, thus compressing further gas bubbles and so on until complete detonation.

Various studies have shown that MS, silicon carbide sand and resin microspheres can enhance the sensitivity to detonation of most EMs. Microspheres and other heterogeneities are regularly added to many suspension and emulsion EMs currently in use, in order to adjust their sensitivity to the desired level, typically high enough for relatively easy initiation. Numerous individual sensitising agents and sensitising agent systems, in line with the above examples, are described in the literature and patents [20,21,22,23,24,25,26]. MS are used as inert sensitising agents for most EMs, including even OSM-type EMs.

As determined in our previous research, MS noticeably accelerate the decomposition of HTP in OSM-type EMs, compared to analogous formulations containing polymer microspheres [18]. The significant increase in the HTP decay rate with MS may be associated with the mechanical shattering of MS, leading to the formation of porous fragments on which HTP can be adsorbed.

## 2. Results

**Figure 1:**
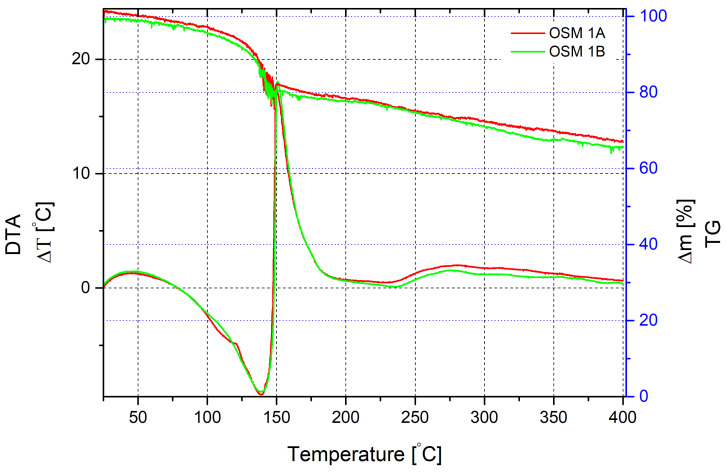
Thermal decomposition of OSM 1A and 1B recorded by DTA/TG. Measurements were conducted in air, at a heating rate of 10 K/min, in the range of 20–400 °C.

The thermal decomposition of OSM 1 was investigated at a heating rate of 10 °C/min (Figure 1), in the range of 20–400 °C.

In the range of 20–80 °C, a weak exothermic effect is observed, accompanied by a slight sample mass loss of the sample. After 80 °C, the reaction becomes endothermic and the mass loss increases, likely due to water evaporation from the mixture. At 120 °C, the active mass loss is accompanied by an endothermic effect down to −9.3 °C, with a peak at 140 °C. The mass loss at this temperature is 15%. Further thermal decomposition is accompanied by rapid heat release, with the peak of the exothermic effect at 150.3 °C, amounting to 17.25 °C. The mass loss at this stage is 19.3%. The significant exothermic effect likely accompanies the chemical interaction of HTP with glycerine. Likely, further mass loss slows down due to the burning of mixture components. The total mass loss up to a temperature of 400 °C is 34.3%.

**Figure 2:**
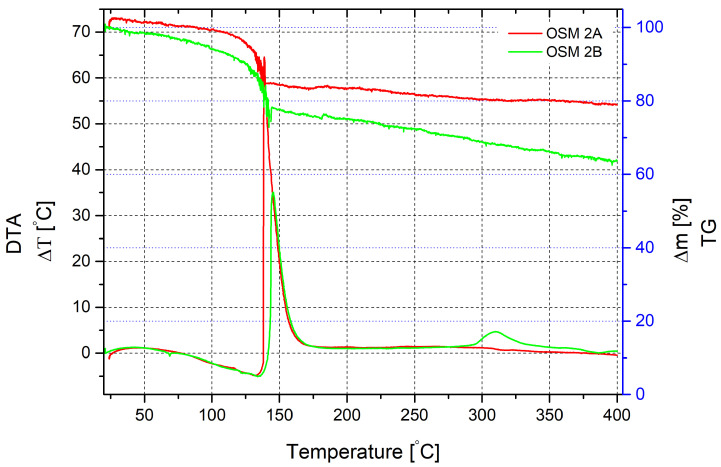
Thermal decomposition of OSM 2A and 2B recorded by DTA/TG. Measurements were conducted in air, at a heating rate of 10 K/min, in the range of 20–400 °C.

Thermogravimetric analysis of the sample using PN and without MS demonstrates practically the same pattern with the difference in a smoothing of a small exothermic effect at a temperature of 122 °C (Figure 2).

According to the nature of the recorded thermograms (unstable linear character), thermal decomposition at the entire temperature range was accompanied by mass exchange processes with the surrounding environment. The oscillations of the mass loss curve on both thermograms can be explained by the decomposition processes of HTP, resulting in the release of oxygen and carbon dioxide bubbles, causing sharp fluctuations in the sample mass.

In conclusion, MS have no impact on the reaction pathways, crucially while using CN as an oxidising agent. It may be due to the formation of a thin layer of poorly soluble calcium compounds on the surface of the MS, leading to the isolation of the glass surface. To further clarify this assumption, microscopic studies of mixtures with the addition of MS were conducted.

Another situation appears in the gradual heating process of the mixture with PN as an additional oxidising agent. Like in the previous scenario, water evaporation and partial decomposition of HTP occur, with mass loss starting from 80 °C. It is accompanied by an endothermic effect on DTA, with a maximum corresponding to a temperature of 132 °C and reaching 5 °C. The primary reaction likely begins at this temperature, accompanied by a characteristic exothermic effect, with a temperature jump reaching 64.5 °C. It corresponds to a heating temperature of 138.7 °C. The mass loss at this point is 15%. The total reaction time is approximately 2 min. Beyond 140 °C, the sample mass continues to decrease monotonously and by 400 °C, the loss is 21% of the initial sample mass.

A similar mixture without MS presents a slightly different pattern. Mass loss starts much earlier but appears more gradual. The peak endothermic effect, similar to the sample with microspheres, occurs at a temperature of 134 °C. The mass loss at this point is 13.5% and continues to increase. At 140 °C, when active heat release begins, it reaches approximately 23%. However, the exothermic effect accompanying the primary reaction for the mixture without microspheres is much smaller than in the previous study. The maximum temperature increase value is only 35 °C. Unreacted substances cause a slight exothermic effect at temperatures of 300–320 °C. Mass loss after the active phase shows a uniform appearance up to 400 °C and reaches 36%.

**Figure 3:**
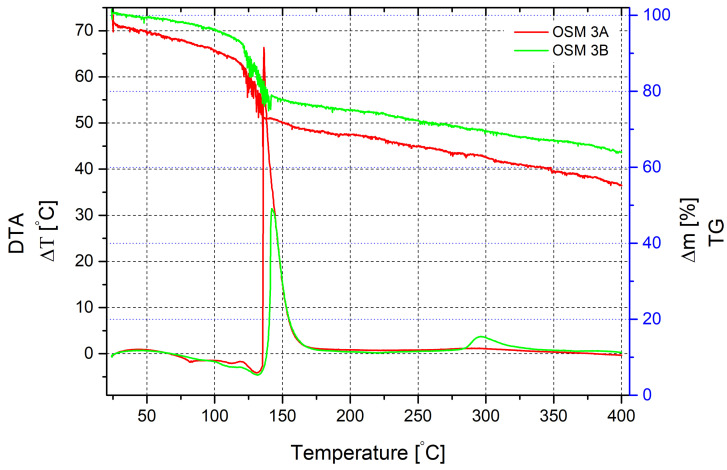
Thermal decomposition of OSM 3A and 3B recorded by DTA/TG. Measurements were conducted in air, at a heating rate of 10 K/min, in the range of 20–400 °C.

Thermogravimetric analysis of the mixture sample containing SN as an additional oxidising agent in the presence of MS demonstrates a pattern similar to the previous sample with PN (Figure 3). Mass loss begins with the onset of heating, appears gradual and is accompanied by a slight endothermic effect at temperatures around 80 °C. After reaching 100 °C, the reaction activates. The maximum endothermic effect, indicating water evaporation and the onset of HTP decomposition, is observed at a temperature of 130 °C. The mass loss at this stage is approximately 21%. We also observe fluctuations in the mass loss curve, indicating active gas evolution, which mechanically affects the device. After reaching a temperature of 135.5 °C, an active reaction begins, accompanied by a significant exothermic effect, with a temperature change reaching 66.3 °C at a heating temperature of 136.2 °C. The mass loss at this point is approximately 27%. Further mass reduction becomes monotonous and at a temperature of 400 °C, it reaches nearly 45%.

The same mixture without microspheres behaves almost identically at the beginning of heating, except for the mass loss, which occurs much more slowly. The active mass loss starts at a temperature of 123 °C and is accompanied by the same fluctuations in the mass loss curve. The exothermic effect begins at 138.5 °C but has a much slower temperature rise and reaches its maximum value at a heating temperature of 142 °C. The peak exothermic effect is 31.5 °C. That is half the value compared to the same mixture with microspheres. The mass loss at this point is 21%. Due to incomplete chemical interaction, another small exothermic effect with a maximum value of 3.5 °C, corresponding to a heating temperature of 295 °C, can be observed. It is not accompanied by fluctuations in the mass loss curve, which becomes monotonous after the primary reaction, reaching 36% at a temperature of 400 °C, nearly 10% lower than in the presence of microspheres. It can be explained by the slowing down of the overall rate of thermal decomposition and the increase in the combustion time of the main mixture components.

**Figure 4:**
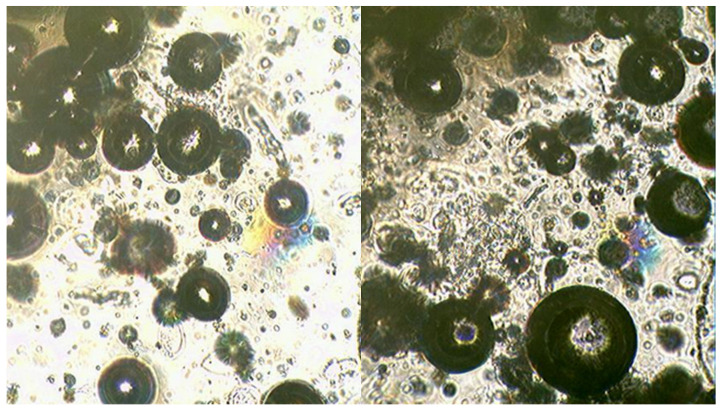
Photographs of OSM 2A PN+MS sample. Microscope Ulab XSP-146T (ULAB Scientific, Nanjing, China), digital camera Sigeta UCMOS 3100 3.1 Mpix (Sigeta, Dembavos, Lithuania) Dembavos. The lens used is 20×.

**Figure 5:**
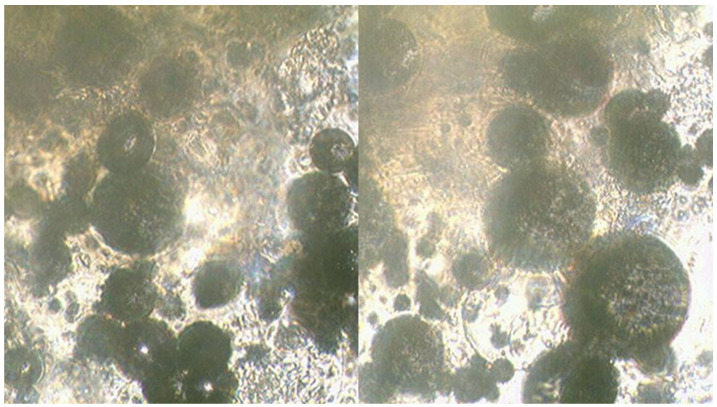
Photographs of the OSM 1A CN+MS sample. Microscope Ulab XSP-146T, digital camera Sigeta UCMOS 3100 3.1 Mpix. The lens used is 20×.

A study of mixtures using the Ulab XSP-146T LED optical microscope was conducted to determine the features of the reaction progress in the presence of PN as an oxidising agent (Figure 4). The results of the microscopic analysis of the mixture containing CN and MS are presented in Figure 5.

MS of clear-cut shape can be observed on the surface where the primary reaction between glycerin, HTP and CN takes place, contributing to a high interaction rate and a significant exothermic effect. Figure 5 shows the microscopic analysis of samples of a mixture containing CN as an additional oxidising agent with the addition of MS.

As can be seen in the figure, the surface of the MS appears uneven, presumably due to the adsorption of sparingly soluble calcium compounds on the surface. However, despite the more developed interaction surface, the reaction rate and exothermic effect in this case are much lower compared to mixtures containing PN and SN as additional oxidising agents.

## 3. Discussion

The research results demonstrate a significant influence of MS on the thermal decomposition processes of OSM EX compositions, particularly those containing potassium nitrate (PN) and sodium nitrate (SN) as additional oxidising agents. The decomposition process of the composition with calcium nitrate (CN) does not depend on a sensitising additive, which is shown on the DTA curves. This effect correlates with previous data on the decomposition of HTP in mixtures during storage, particularly, that CN, even in the presence of MS, significantly inhibits the HTP decomposition process and is the only additive under which the effective concentration of HTP remains above 30% mass for a day.

The influence of MS on the rate and completeness of decomposition can be partially explained by the density dependence of the samples, which directly affects their detonation velocity (VoD). Mixtures containing MS have noticeably higher densities, leading to a comparable difference in VoD values for these mixtures. However, it does not explain the absence of any influence from adding microspheres to the composition with CN. In this case, according to the mentioned hypothesis, there should be a noticeable impact since there is a sufficient density difference between the OSM 1A and OSM 1B samples. However, this does not occur.

MS are always added to EMs as inert sensitising agents [27]. Their mechanism of influence is usually explained by increasing the reaction area and creating local heating centres during detonation, as per widely reported mechanisms—these heating centres are typically referred to as “hot-spots” [28]. As revealed in this study, this is not entirely the case with OSM. MS significantly accelerate the decomposition of HTP, as shown in a previous study. HTP decomposition activates the chemical interaction and increases the VoD. However, such behaviour cannot be explained solely by increasing the active surface due to the destruction of MS, which leads to porous fragments’ formation, capable of adsorbing HTP. As seen from the results of the microscopic investigation of mixture OSM 1B, MS in the composition have a much more developed surface, likely due to adsorption on their surface of sparingly soluble calcium compounds, but this does not affect the detonation process. Evidently, there is a chemical interaction between the composition and the surface of MS, which is absent in the case of using CN. The mechanism of this interaction is unclear, even though this phenomenon has been observed in the literature and is typically minimised via rinsing glass vessels, designed to hold HTP, sequentially with hot nitric acid, distilled water and, finally, with HTP. This suggests that the Si-O^−^ groups present on the surface of glass may promote HTP decay, as rinsing with nitric acid will result in those groups being protonated, forming silanol (Si-OH) functionalities [29]. Another possible source of this issue is the presence of trace amounts of catalytic impurities (e.g., alkali metals, iron cations) in the microspheres. Therefore, further research is necessary to establish the interaction mechanism between mixture components and the possible catalytic influence of MS components.

As for the choice of oxidising agent, any oxidising agent other than CN may be considered, given the insignificant difference in the energetic characteristics of the OSM 2A and OSM 3A mixtures. A preference may be given to a mixture with higher density [18] to maximise the VoD of the explosive material. Considering that the OSM 1A mixture, which has the highest density value, is not optimal according to the research results, composition OSM 2A will be considered optimal.

## 4. Materials and Methods

### 4.1. Materials

The used components are presented in Table 1. All components were used as received, without any purification.

The explosive mixture samples consisted of an HTP and glycerine, to which CN, PN and SN were added as additional oxidising agents. GG was used as a thickener. Samples were prepared with the addition of a sensitising additive—GM—and without them. MS of type K015 are hollow glass spheres with a typical density of 0.15 g/cm^3^ and an isostatic crushing strength of 300 psi.

### 4.2. Preparation of Mixture Samples

**Figure 6:**
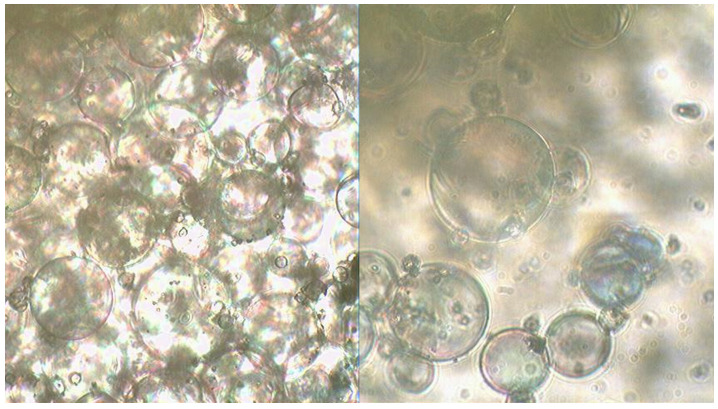
Samples of MS type K-015 (MS). Microscope Ulab XSP-146T, digital camera Sigeta UCMOS 3100 3.1 Mpix. The lens used is 20×.

First, the auxiliary oxidising agent (SN, PN, or CN) was premixed with HTP using a mechanical stirrer made of inert material at a speed of 200 rpm at room temperature until the nitrate was completely dissolved. After reducing the stirring speed to 100 rpm, glycerine and guar gum were added, followed by a 10-minute mixing and homogenisation cycle. Samples were prepared in equipment made from polymers to avoid the catalytic effect of the metals and impurities that microspheres may contain. Thus, almost any type of contamination can increase the rate of HTP decomposition and in some extreme situations, it can lead to a detonation event. Then, MS were added and gently mixed to minimise the damage. Even before preparing the OSM compositions, the MS contained a fraction of broken microspheres, which can be seen in Figure 6.

Samples were obtained at a constant temperature in dark conditions and loaded into identical containers made of inert material. It took 5 min to prepare each sample. The thermogravimetric analysis started 5 min after preparing the mixture sample. Samples weighing 0.2 g were taken and placed in a crucible and the thermal decomposition process was investigated using a derivatograph, previously set to the operating mode. The composition of the researched samples is provided in Table 2, and lifetimes recorded for the previously studied OSM formulations are given in Table 3.

### 4.3. Thermal Analysis

Thermogravimetric research was conducted using a Thermoscan-2 derivatograph (Analitpribor, St. Petersburg, Russia) for samples weighing 200 mg, with a heating rate of 10 K/60 s for all samples. Experiments were carried out in ambient air in all cases. The change in heat content of the substance with temperature was recorded based on measuring the temperature difference between the research sample and a reference sample, in which no transformations occurred within the investigated temperature range (20–400 °C). The change in sample weight with temperature was determined using an electronic continuous weighing system.

### 4.4. Microscopic Investigation

Microscopic investigations were conducted using a Ulab XSP-146T LED microscope (ULAB Scientific Instruments Co., Ltd., Nanjing, China), illuminated with Keller lighting and equipped with a Sigeta UCMOS 3100 3.1 MP digital video camera. In standard configuration, the microscope is designed for bright-field microscopy in transmitted light. This modification of the microscope has built-in bottom LED illumination. The Sigeta UCMOS 3100 3.1 MP digital camera is an eyepiece digital camera for microscopes with a 3-megapixel resolution, specifically designed for high-resolution imaging of the investigated object on a computer. All measurements were performed in backscattering geometry using a microscope objective with 20× magnification and a numerical aperture of 0.4, which, in combination with the WF10× eyepiece and camera, provides a total magnification of 200×.

### 4.5. Safety

The described samples are explosive in nature and after preparation, particular care should be given to handling them. Since these are semi-liquid in form, the materials exhibit limited sensitivity to mechanical stimuli. Other types of initiating stimuli should, however, be strictly avoided, particularly exposition of the samples to heat. An important consideration for handling the OSM-type EMs is that the addition of any impurities must be carefully avoided, as such impurities will influence the decomposition dynamics of HTP within the samples, leading to significant changes in their properties.

## 5. Conclusions

Multiple OSM-type EM formulations, utilising various auxiliary oxidising agents and either containing or lacking sensitising agents (glass microspheres), have been investigated in terms of thermally-induced decomposition. It was found that the presence of microspheres promotes the thermal decomposition of all OSM samples, across a wide range of temperatures, with the effect being particularly pronounced at temperatures above the DTA peak temperature. This is likely due to the interaction between the microsphere surface and HTP.

An interesting feature of the investigated samples is their relative resistance to heating to temperatures of at most 100–120 °C, as at these temperatures, only a minor influence of temperature on the rate of mass decrease in the sample is observed. Moreover, instead of a phase transition signal (e.g., due to the boiling of water), an exothermic decomposition peak is observed at approx 150 °C for all the investigated formulations.

In terms of the choice of auxiliary oxidising agent, while CN appears to have disadvantages in terms of thermal properties (slight exothermic peak at approx 122 °C), both SN- and PN-based OSM formulations appear to exhibit very similar thermal properties.

The influence of MS on the rate and completeness of decomposition can be partly explained by the density dependence of the samples. Their influence mechanism is explained by the increase in the reaction area and the creation of local heating centres during detonation [30].

## Figures and Tables

**Table 1:** Materials used in this work.

Chemical (Code)	Purity Grade	Source
Sodium nitrate (SN)	>99%	Chempur (Piekary Śląskie, Poland)
Potassium nitrate (PN)	>99%	Chempur (Piekary Śląskie, Poland)
Calcium nitrate tetrahydrate (Ca(NO_3_)2·4H_2_O) *	>99%	Chempur (Piekary Śląskie, Poland)
Hydrogen peroxide 60 wt.% (HTP)	analytical	Chempur (Piekary Śląskie, Poland)
Glycerine	>99.5%	TechlandLab (Tarnobrzeg, Poland)
Guar gum S.C.–406 (GG)	>99%	Meyhall Chemical AG (Kreuzlingen, Switzerland)
Glass microspheres type K-015 (MS)	n/a	3M (Saint Paul, MN, USA)

* The reagent was dried in 373 K for 24 h before use, to remove the hydration water.

**Table 2:** The composition of the studied samples, in wt.%.

OSM: *	OSM 1A	OSM 1B	OSM 2A	OSM 2B	OSM 3A	OSM 3B
SN	-	-	-	-	9.9	9.9
PN	-	-	9.9	9.9	-	-
CN	9.9	9.9	-	-	-	-
GG	3	3	3	3	3	3
HTP	71	71	71	71	71	71
Gl	15.1	15.1	15.1	15.1	15.1	15.1
MS	1	-	1	-	1	-

* The composition is the sum of the components listed in the corresponding column.

**Table 3:** Comparison of the time required for the concentration of hydrogen peroxide in the OSM samples to decrease to a fourth of the initial measured concentration. Adatped from [18]. It should be noted that OSM codename schemes were altered, due to the omission of ammonium nitrate-based OSMs in this study.

OSM:	OSM 1A	OSM 1B	OSM 2A	OSM 2B	OSM 3A	OSM 3B
T_1/4_ [h]	12.32	-	8.77	-	12.76	-

## Data Availability

The data presented in this study are available on request from the authors.
